# An evanescent mass found in the transthoracic echocardiography: microcavitation phenomenon

**DOI:** 10.1093/ehjcr/ytae400

**Published:** 2024-08-07

**Authors:** José Antonio Fernández-Sánchez, Ismael Arco-Adamuz, Torcuato Garrido-Arroquia Jurado, José Manuel Oyonarte-Ramírez

**Affiliations:** Cardiology Department, Hospital Universitario Virgen de las Nieves, Av. Fuerzas Armadas 2, 18002 Granada, Spain; Cardiology Department, Hospital Universitario Virgen de las Nieves, Av. Fuerzas Armadas 2, 18002 Granada, Spain; Cardiology Department, Hospital Universitario Virgen de las Nieves, Av. Fuerzas Armadas 2, 18002 Granada, Spain; Cardiology Department, Hospital Universitario Virgen de las Nieves, Av. Fuerzas Armadas 2, 18002 Granada, Spain

## Case description

A 61-year-old female patient came to the outpatient clinic for her annual follow-up.

Her past medical history included atrial fibrillation and rheumatic heart disease with a mechanical mitral valve replacement and tricuspid annuloplasty back in 2002.

A transthoracic echocardiography was performed showing a normal left ventricle with nearly normal ejection fraction (52%), a severe biatrial enlargement, subvalvular surgical remnants, and mitral prosthesis with no signs of dysfunction and both discs moving appropriately (see Supplementary material online, *Videos S1 and S2*).

**Figure 1:**
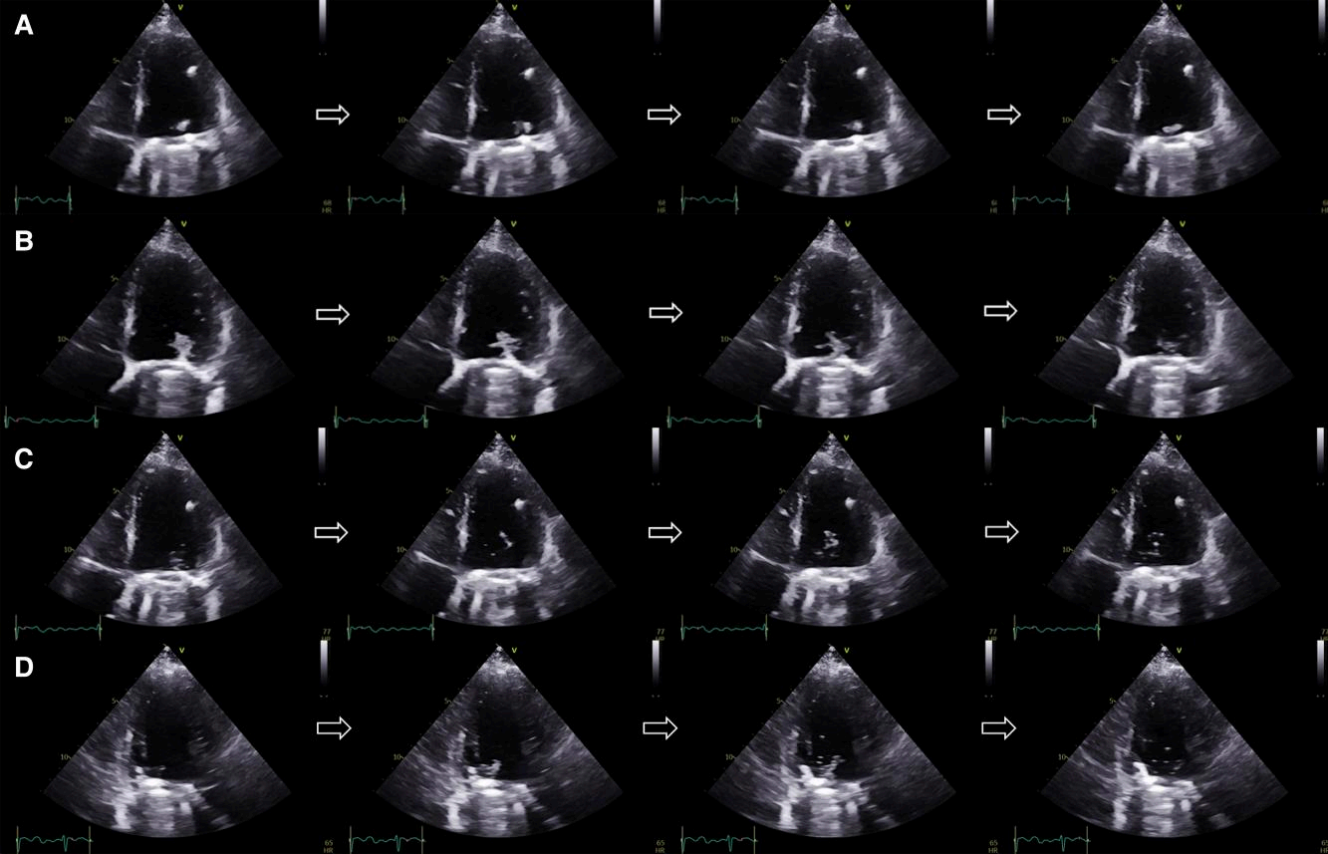
Transthoracic echocardiogram. (*A–C*) Apical four-chamber. (*D*) Apical three-chamber. (*A*) A mobile, echo dense mass in the ventricular surface of the mechanical mitral valve. (*B*) Changes in the size and shape of the mass. (*C* and *D*) The cluster of microbubbles and how they disappear.

However, on the ventricular surface of the prosthesis, lateral to the annulus, a mobile echo dense image resembling a mass was observed (*Figure 1A* and *B*; Supplementary material online, *Videos S3* and *S4*). The initial images were reviewed in slow motion, and it was noticed that as the cardiac cycle went forward, the ‘mass’ started to disintegrated into microbubbles (*Figure 1C* and *D*; Supplementary material online, *Videos S5* and *S6*). Due to these features and the evanescent behaviour, microcavitation phenomenon was diagnosed.

This phenomenon happens when blood travels through the narrowest portion of the prosthesis and local pressure falls quickly under evaporation pressure conditions forming microbubbles. Then, in the ventricle, the pressure recovery forces the gas to condense back and microbubbles disappear.^1^

Although it is a rare find, it is essential to be familiar with it in order to avoid unnecessary interventions as it could be confused with other echo dense lesions such as strands, surgical remnants, and, more importantly, masses like vegetation or thrombus.^2,3^

Incorporating the clinical findings along with the observation of a hypermobile and quickly disappearing mass (with changes in size and shape in different images) may help to resolve the imaging dilemma. Additionally, modifying or even turning off the harmonics may be helpful although sometimes it can be a real challenge that will require more advanced imaging techniques.

## Supplementary Material

ytae400_Supplementary_Data

## Data Availability

The data underlying this article are available in the article and in its online Supplementary material.

## Supplementary material

**Consent:** The patient gave his consent to appear in the publication, in accordance with the COPE guidelines.

**Ethics approval:** Our institution does not require ethical approval for reporting individual cases or case series.

**Funding:** None declared.
